# Review of antibiotic-resistant bacteria and antibiotic resistance genes within the one health framework

**DOI:** 10.1080/20008686.2024.2312953

**Published:** 2024-02-13

**Authors:** Ayodele Oluwaseun Ajayi, Adebowale Toba Odeyemi, Olajide Joseph Akinjogunla, Akinwole Babafenwa Adeyeye, Ibiwumi Ayo-ajayi

**Affiliations:** a Department of Microbiology, Federal University Oye Ekiti, Ekiti State, Nigeria; b Department of Microbiology, Landmark University SDG Groups 2 and 3, Omu-Aran, Kwara State, Nigeria; c Department of Microbiology, Faculty of Science, University of Uyo, Akwa-Ibom State, Nigeria; d Department of Computer Science, Afe Babalola University, Ado Ekiti, Ekiti State, Nigeria

**Keywords:** One health, antibiotic resistant bacteria, antibiotic resistance genes

## Abstract

**Background:** The interdisciplinary One Health (OH) approach recognizes that human, animal, and environmental health are all interconnected. Its ultimate goal is to promote optimal health for all through the exploration of these relationships. Antibiotic resistance (AR) is a public health challenge that has been primarily addressed within the context of human health and clinical settings. However, it has become increasingly evident that antibiotic resistant bacteria (ARB) and antibiotic resistance genes (ARGs) that confer resistance are transmitted and circulated within humans, animals, and the environment. Therefore, to effectively address this issue, antibiotic resistance must also be considered an environmental and livestock/wildlife problem.

**Objective:** This review was carried out to provide a broad overview of the existence of ARB and ARGs in One Health settings.

**Methods:** Relevant studies that placed emphasis on ARB and ARGs were reviewed and key findings were accessed that illustrate the importance of One Health as a measure to tackle growing public and environmental threats.

**Results:** In this review, we delve into the complex interplay of the three components of OH in relation to ARB and ARGs. Antibiotics used in animal husbandry and plants to promote growth, treat, and prevent infectious diseases lead to the development of antibiotic-resistant bacteria in animals. These bacteria are transmitted from animals to humans through food and environmental exposure. The environment plays a critical role in the circulation and persistence of antibiotic-resistant bacteria and genes, posing a significant threat to human and animal health. This article also highlights how ARGs are spread in the environment through the transfer of genetic material between bacteria. This transfer can occur naturally or through human activities such as the use of antibiotics in agriculture and waste management practices.

**Conclusion:** It is important to integrate the One Health approach into the public health system to effectively tackle the emergence and spread of ARB and genes that code for resistance to different antibiotics.

## Introduction

An interdisciplinary approach called ‘One Health’ involves the collaborative effort of different sectors aimed at providing optimal health for all by recognising the close relationship between the health of humans, animals and the environment [1]. The concept of one health has been recognized for a long time [2]. Although One Health is not defined in a single, universally accepted manner, all the definitions provided by different agencies and bodies recognize that ecological health, animal health, and human health are all connected [3]. According to the University of California at Davis’ One Health Institute, One Health is a strategy for ensuring the wellbeing of people, animals, and the environment through cooperative problem solving on a local, national, and international level [4]. However, the concept became more popular in recent years due to some emerging global problems like climate change, astronomical population growth, upsurge in emerging infectious diseases, antimicrobial resistance and deforestation, among others [5–8]. The principle of One Health further emphasizes that occurrences in natural ecosystems at the animal and environment interface have a profound influence on human health [9]. This buttresses the fact that several public health problems can be tackled at the periphery of One Health and that these approaches are cheaper for tackling such problems compared with other core clinical and public health approaches [4,10]. According to the World Health Organization, animals, both domestic and wild, are the source of over 60% of new infectious diseases that are reported globally. The previous three decades have seen the discovery of over 30 novel human diseases, 75% of which have animal origins [11].

### Global efforts for One Health

This emergent view has made it mandatory for different governments and institutions across the globe to prioritize One Health and incorporate the framework into their national and policy health frameworks to achieve their cardinal public health objectives [12,13]. The African Ministers of Health signed a One Health declaration and later approved an action plan to increase health and environmental actions over a ten-year period to enhance public health by the year 2030 [14]. Furthermore, the major global institutions like the World Health Organisation (WHO), Food and Agriculture Organisation (FAO) and the Organisation for animal Health (OIE), initiated a joint alliance to fight antimicrobial resistance through the One Health approach. The goal of this alliance is to assist member countries to achieve their respective One Health initiatives and define a global framework for One Health [11]. Some innovative surveillance platforms have also been developed to serve as tools for surveillance of ARB and ARGs. Such tools include the Global Antibiotic Resistance Surveillance System (GLASS) developed by the WHO, and provides an integrated surveillance approach globally and for different countries. Different bioinformatics and metagenomics workflows and databases have also been developed to assist in surveillance of antibiotic resistance genes in different environments [15]. The outbreak of emerging infectious diseases and pathogens like the severe acute respiratory syndrome (SARS), avian influenza virus disease, and the most recent COVID-19 caused by the SARS-Cov2, which has put the world in a tight spot, has drawn the attention of many countries, agencies, and institutions to the irrefutable fact that many pathogens that affect human life, wellbeing, and trade can emerge from wildlife, and these animals have continued to share the same ecosystems with humans [16]. Another institutional mechanism to limit the use of antibiotics in foods and animals in order to lower the risk of the emergence of ARB is the Maximum Residue Limit (MRL) fixed by JECFA of CODEX. The MRL is the maximum concentration of residue legally tolerated in a food product obtained from an animal that has received veterinary medicine, including antibiotics. The adoption of the MRL for specific antibiotics in animals and meat products indirectly imposes safety limits on such animal products [17].

### Transmission pathways and threats

The emergence of zoonotic diseases is an integral aspect of One Health [18,19]. The transmission of zoonotic bacterial pathogens, virulence and resistance genes, and diseases across national and international boundaries is facilitated by the mass movement of animals from one country to another, enhanced by modern, rapid, and sophisticated transportation networks [20,21]. These zoonotic diseases may be caused by bacteria, viruses, or parasites, with animals playing a vital role in maintaining and transmitting such infections. Over the past decade, the diminishing delineation of the human and animal ecosystems (wildlife) due to massive industrialisation, deforestation, and other severe anthropogenic events have drastically increased the interface or contact between animals and humans [20,22]. The clinically relevant bacteria that have also been designated as critical pathogens have emerged as important aspects of zoonoses. For example, *Acinetobacter baumannii* is a common nosocomial pathogen and has also emerged as a leading zoonotic bacterium. *Salmonella typhi* is the leading cause of typhoid fever in sub-Saharan Africa, and the endemicity of the disease is partly due to the widespread presence of the bacterium in livestock and poultry [23]. This is also responsible for the difficulty to effectively control the spread and prevalence of the disease in the regions or populations affected by the disease [24]. *Pseudomonas aeruginosa* is another critical bacterial pathogen that has also evolved and disseminated from domestic animals and has raised serious concern concerning its possible dissemination to humans, together with its high virulence potential [25]. Several bacteria with proven resistance to antibiotics considered relevant in clinical and veterinary settings carry ARGs that code for resistance to such bacteria. The presence of ARGs in different environments qualifies them as environmental pollutants and their impact within the human-animal-ecosystem interface qualifies them for One Health. Animals carry ARGs and disseminate them to the ecosystem and such genes can be biomagnified in associated environments.

These increased interfaces have been closely linked with the upsurge in the rate of emerging infectious diseases like COVID-19 and the occasional upsurge in the prevalence of other infections like Lassa fever [26]. Another common phenomenon is the disruption of the wildlife and animal ecosystems that has led to the encroachment of residential areas by animals, further increasing the risk of transmission of emerging pathogens that are naturally a component of the wildlife [27,28]. Other external threats can also be factored into One Health, and the most important is climate change [29,30]. The increasing threat to the global ecosystem due to rising global temperatures has been recognized as a serious risk factor for the emergence of novel bacterial, fungal and viral pathogens either on the epidemic or pandemic scale [31,32]. It also makes wild life less conducive for animal habitation, with an associated increase in the encroachment of animals into humans’ ecosystems, and vice versa [33,34]. Climate change has set a strong precedence for increased risk of susceptibility to infectious diseases among animals and humans [31,35].

The cumulative of all these factors buttress the importance of the One Health initiative to tackle global health problems at this crucial interface. Several other environmental factors within the One Health paradigm have also been recognised to affect human and environmental health. The massive human activities and anthropogenic events have been linked with the release of extraneous factors that adversely affect the environment, which in turn affects animals and humans. Such factors include drugs, chemicals, and antibiotic residues that emanate from manufacturing plants [36–38]. Biological wastes from the agricultural and food industries are also important sources of environmental pollutants. The release of these hazards can also be found in water bodies and waterways, with attendant risks to public health [36,37].

In view of the importance of the One Health framework in the control of antibiotic resistance, dissemination of ARGs, and associated public health threats, this review seeks to provide an overview and a basic understanding of the threats posed by the emergence of antibiotic-resistant organisms and ARGs properly situated within the One Health framework. It also provides a justification for the study of these threats within the One Health framework, with an emphasis on how the emerging threats can be investigated and controlled.

## Antibiotic resistance within one health framework

Antibiotic resistance is recognized globally as a One Health issue and has been well situated within various One Health policy frameworks in different nations [5,39]. This implies that the problems associated with antibiotic resistance can be tackled using the One Health policy approach [40]. Numerous studies have demonstrated that the One Health approach is a more cost-effective choice that can be used to tackle the issue of antibiotic resistance to mitigate and control the transmission of antibiotic-resistant bacteria and antibiotic-resistant genes when compared to other conventional approaches that have been implemented to address the issue [6,41].

Historically, antibiotic resistance was initially positioned within the human health context and clinical settings [42,43]. As a matter of fact, antibiotic resistance was first observed among humans within clinical environments before reports emerged of antibiotic resistance among humans in community settings. There are extensive research and development that has led to the discovery of antibiotics that are subsequently used for treating infectious diseases in humans [42]. Penicillin, tetracycline, streptomycin, and other beta-lactam antibiotics were among the first to be identified and deployed for use in humans [43,44]. There is evidence of the effectiveness of antibiotics against bacterial pathogens in humans and such pathogens later developed resistance against the first line of antibiotics that were used [44].

In addition to the facts already provided, the use of antibiotics in humans is not only limited to clinical settings, they are also used extensively by larger populations in community and related extra-clinical settings [45]. Antibiotics have become the mainstay of modern medicine and are crucial for the successful outcome of various healthcare procedures. There has been documented application of antibiotics in the treatment of bloodstream infections [46], urinary tract infections [47], skin and soft tissue infections treated with dicloxacillin, cephalexin/trimethoprim plus sulfamethoxazole [48], and sexually transmitted infections [49].

In addition to the application of antibiotics in the management of active infections, their prophylactic use in preventing infections is equally well documented [50]. The most common use of antibiotics for prevention is surgical prophylaxis, where antibiotics are administered to prevent infections and complications during surgical procedures [51]. The first-generation cephalosporins have been the most commonly used antibiotics for surgical prophylaxis. Surgical site infections are the most common infections reported to require antibiotic prophylaxis. The prophylactic use of antibiotics, especially in human and veterinary settings carries increased risk of resistance to antibiotics [52].

The misuse and abuse of antibiotics have been linked to the high prevalence of antibiotic-resistant bacteria within hospital settings [53]. This problem has strengthened various policies that promote antimicrobial stewardship in healthcare settings, lessen the impact of antibiotic resistance, and mitigate the risk of transmission of antibiotic-resistant bacteria and their resistance genes from the clinical setting into the community and environment [54]. Equally important is the use of antibacterial drug use in veterinary contexts, even though the emphasis has historically tilted towards antibiotic use in human medicine [55]. One of the major aims of the One Health strategy concerning antibiotic resistance is the holistic consideration of the problem in order to deal with antibiotic resistance using an integrated approach. Within this purview, the veterinary use of antibiotics also demands adequate attention to be able to situate the multidimensional scope of the problem posed by antibiotic resistance. Antibiotics are utilised in livestock to treat infections that are known to increase animal mortality and morbidity, which further reduce yield and agricultural productivity [56]. Tetracycline is frequently used for the treatment of avian illnesses, especially in low resource countries while amoxicillin is widely used in swine farming [57]. The use of last-resort antibiotics, such as colistin, which has been advised to be used only in human medicine, is more worrisome [58]. Apart from their use in poultry, the tetracycline family is also widely used for different purposes in food animals, especially among livestock and aquaculture [56,59]. The use of antibiotics for prophylactic treatment of animals also features significantly [60]. There have been estimates that the quantity of antibiotics used for prophylaxis among animals slightly outweighs the quantity used to treat active infections in animals [61].

In addition to being used to treat and prevent infections, antibiotics are also used for growth promotion. This aspect of antibiotic usage is believed to have contributed significantly to the overall increase in the quantity of antibiotics in agriculture [62]. The overriding aim of commercial agriculture is to increase profit and productivity, which is secondary to the process of raising animals for human consumption, and the use of antibiotics for growth promotion appears to be central to this aim. One of the most common examples of the use of antibiotics for growth promotion is the in-feed addition of tetracycline, colistin, and macrolides [63]. In addition to the use of antibiotics to boost growth, other inorganic compounds also have a common use as alternatives to antibiotics as growth promoters. Such compounds include zinc bacitracin, enramycin, halquinol, virginiamycin, and avilamycin [64]. Another dimension to the use of antibiotics is their application to treat infections, administer prophylaxis, and possibly enhance growth in plants [61]. The most common antibiotics used in plants are tetracycline and streptomycin [65,66]. These two antibiotics are also critical for their different uses in clinical environments. Streptomycin is one of the first-line drugs for the treatment of tuberculosis, while tetracycline has a wide array of uses for the treatment of respiratory tract infections and urinary tract infections [67]. The global prevalence of tuberculosis, caused by *Mycobacterium tuberculosis*, has been increasing over the years, especially in low- and middle-income countries. This is partly due to the zoonotic origins of some strains of the bacterium that are mostly resident in cattle, pigs, and other livestock. The frequent interface of farmers with their livestock increases the risk of contracting the bacterium, with attendant effects on public health [68].

In order to situate these phenomena within the One Health framework, the cumulative effect of the various uses of antibiotics in human healthcare, animals, and plants is the deliberate release of small and unintended doses of residual antibiotics directly into the environment [69]. More specifically, the direct application of antibiotics to plants results in the discharge of small antibiotic pollutants into the soil [69]. Another significant aspect of environmental antibiotic contamination is the release of antibiotic pollutants into water bodies due to runoff caused by heavy rainfall following antibiotic use.

Additionally, several practices related to immigration also increase the likelihood of the release of residual antibiotics present in the soil into adjoining waters. Significant occurrences of residual antibiotics have been found in water bodies and aquatic environments through the application of antibiotics to aquaculture and heavy runoff of residual antibiotics from the soil [70]. All these interrelated phenomena can be resolved within the One Health framework. Equally complicating this concern is the emergence of antibiotic resistant bacteria from humans, animals, and plants that are also released into the environment.

It is on this premise that the existence of microorganisms that are resistant to antibiotics, and their associated antibiotic-resistant genes have been designated as an environmental issue that can be positioned within the One Health framework [71]. Three major domains of antibiotic resistance have been clearly identified as follows:

- The emergence of antibiotic-resistant bacteria from humans,
- The emergence of resistant bacteria from animals and
- The emergence from the environment and subsequent release of such bacteria from animals and humans into the environment. (Chang *et al*., 2015).

There have been numerous reports confirming that antibiotic-resistant bacteria that emerge from core healthcare settings have been disseminated into the community, and this further imperils the health of the population [72]. Furthermore, the WHO global bacterial resistance surveillance system (GLASS) is published every year with the last edition published in 2022 [73]. According to the report, The most common categories of antibiotic-resistant bacteria that are common within the hospital environments include extended spectrum beta-lactamase-producing *E. coli* and *Klebsiella pneumoniae*, responsible for urinary tract and blood stream infections; vancomycin-resistant enterococci that have been identified in bloodstream infections; methicillin-resistant *Staphylococcus aureus*, responsible for bloodstream infections and skin and soft tissue infections; *Salmonella* and *Shigella* spp. responsible for gastroenteritis and show resistance to ciprofloxacin and carbapenem-resistant *Acinetobacter baumannii*, which is an emerging threat within the hospital environment. The clonality of some of these clinically relevant bacteria has been established in clear association with their environmental counterparts. Some strains of *Pseudomonas aeruginosa* have been found to be related to strains associated with the environment [74]. It has also been confirmed that certain clonal types of ESBL-producing *E. coli* have been simultaneously found among humans, foods, and environments [75].

Other common antibiotic-resistant bacteria include non-typhoidal salmonella, *Pseudomonas aeruginosa*, multidrug-resistant *Mycobacterium tuberculosis*, and *Enterobacter aerogenes* [76–78]. The emergence of bacteria that resist several different types of antibiotics adds another layer of complexity to the growing burden of antibiotic resistance. This phenomenon reduces the options for antibiotics that are available as a means of treating bacterial infections within clinical environments [79]. They have been associated with prolonged stays within the hospital environment and have increased the rate of treatment failure among patients with such infections [80].

The transmission and dissemination of ARB from healthcare settings into the community have been widely documented [81,82]. Antibiotic-resistant bacteria can spread from the healthcare setting to the community through a variety of channels. Healthcare workers are most at risk of contracting and spreading ARB from hospitals and related healthcare settings to the community [83]. Inanimate objects have also been identified as agents for the transmission of community-wide antibiotic-resistant bacteria. Additionally, hospital visitors could serve as transient carriers of antibiotic-resistant bacteria within the community. Community carriage has also been linked with healthcare-associated infections among patients who received treatment in hospitals [84].

As previously mentioned, antibiotic use for therapeutic and prophylactic purposes in animals is also a significant factor in the development of bacteria and other organisms resistant to antibiotics. Such ARB are constantly shed into the immediate environment during defecation [85]. Depending on the pharmacokinetics of the specific antimicrobials, residual concentrations of antibiotics used for animals, especially poultry and livestock, are excreted from the animals, and these residual concentrations of antibiotics found in soil and water create a selective pressure in the environment that promotes the emergence of environmental bacteria that show resistance to the respective antibiotics [86]. ARB within the environment have found their way into the guts of animals and fish on land and in water, respectively. As highlighted earlier, extensive runoff can occur during rainfall and erosion that will transfer bacteria that are resistant to antibiotics from the soil to the aquatic ecosystem. All these highlighted factors show the integrated aspects of resistance to antibiotics within One Health and the heightened risk of the transfer of bacteria resistant to antibiotics from animals and the environment to humans, with consequent effects on general public health [87].

Another significant problem of AR within the One Health framework is the emergence of bacteria that show reduced susceptibility to last-resort antibiotics, especially colistin and carbapenems, as well as other clinically significant antibiotics [88]. There are documented cases of colistin use among livestock. This antibiotic is a member of the polymyxin family, and it is usually administered when other alternatives have failed or proven to be less effective [89]. The resistance to this antibiotic and other last-resort antibiotics that have emerged from animals has been found in different environments, and this increases the potential for transmission of bacteria that show resistance to these last-resort antibiotics into the human population. Such a situation has heightened the crisis of antibiotic resistance. Some countries have responded to this emergent crisis by banning clinically relevant last-resort antibiotics in animals [90].

## Antibiotic resistance genes and one health

**Figure 1.**
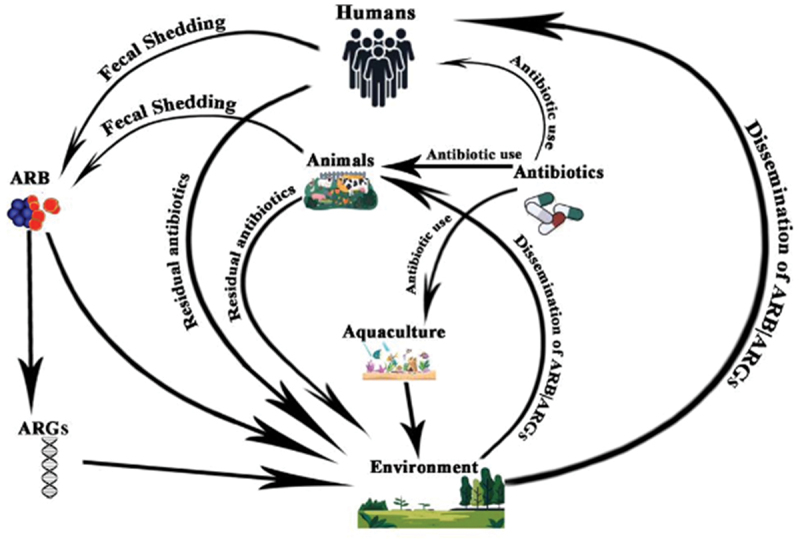
Schematic diagram for dissemination of ARB/ARGs in one Health.

ARGs have been designated as an emerging environmental pollutant and constitute an environmental problem [91,92]. The proper designation of the antibiotic resistance genes within ecosystems as an emerging environmental problem allows them to be situated within the One Health framework in view of the various sources of antibiotic resistance genes that disseminate or transmit into different environments [5,93]. The common sources of antibiotic resistance genes that are disseminated into the environment include domestic or wild animals [94], clinical sources (which include humans and clinical effluents containing antibiotics), and associated resistance genes), and various anthropogenic factors that include industrial production of antibiotics and agricultural uses of antibiotics [95]. The schematic illustration is shown in Figure 1.

The extensive antibiotic use in animals selects antibiotic-resistant bacteria through different genetic mechanisms [36]. Several genes and genetic elements that confer resistance to several antibiotics like ampicillin, streptomycin, sulfamethoxazole, and tetracycline have since been found in animals [96]. The most common genes for antibiotic resistance that are reported in animals include genes that code for resistance to tetracyclines (*tet*), sulfonamides (*sul*), *β*-lactams (*bla*), macrolide-lincosamid-streptogramin B (MLSB) (*erm*), aminoglycosides (*aac*), FCA (fluoroquinolone, quinolone, florfenicol, chloramphenicol, and amphenicol) (*fca*), colistin (*mcr*), vancomycin (*van*), and multidrug (*mdr*) [97]). Furthermore, several genes that confer resistance to antibiotics among bacteria found in animals are situated on plasmids and other forms of mobile genetic elements. These genetic elements are prolific in their ability to replicate and spread ARGs within different ecosystems [98,99]. When such genetic elements are disseminated into the environment alongside the bacteria that carry them, they are further spread in the environment using the intricate molecular mechanisms that enable the antibiotic resistance genes to spread in the immediate environment. As a result, different ARGs have been reported to have been spread into the environment through livestock, poultry, aquaculture, and other intensive and large-scale agricultural practices [100].

Across the globe, large-scale poultry farming meets the growing protein needs of millions of people. The majority of poultry and poultry products produced are produced through intensive and large-scale poultry farming, with economic estimates running to 380 billion dollars [101]. In addition to the predominance of ARGs in poultry feces, many related poultry products also have a huge dose of ARGs. ARB have been found in poultry feeds, drinks, meat, and skin [98,102]). The presence of ARB and ARGs in the adjoining poultry environments is commonplace and has significantly raised serious concern regarding the biosafety of the poultry production process. This is a typical issue that can be addressed by One Health [103].

The meat and milk from livestock, particularly cattle and ruminants, are important sources of protein for populations globally, with an estimated net worth of 1.4 trillion dollars [104]. There is a current emerging trend in the discovery of ARB and ARGs in milk, milk products, and dairy environments [105]. Many ARGs have also been found in bovine milk and the related environment and production facilities [106]. Different metagenomics tools have been used to study livestock and agricultural environments, and some of these studies have shown that the dairy environment has served as a source of ARGs in dairy products from dairy production plants [107].

In addition to the numerous documented roles of plasmids in the emergence and transmission of ARGs, several types of mobile genetic elements (MGEs) have been implicated for their roles in the evolution of ARB and dissemination of ARGs, cutting across different environments and niches. These genetic elements possess the inherent capability to move or transpose into different genomic locations among bacteria [108]. Such MGEs include transposons, insertion sequences (IS), prophages, integrons, and pathogenicity islands [109,110]. Transposons are short pieces of genetic elements that can transpose between plasmids and bacterial chromosomes, and they have been confirmed to carry antibiotic resistance genes [111,112]. The IS have been confirmed to carry specific antibiotic resistance genes, which include *catA1* (chloramphenicol), *tetA* and *tetB* (tetracycline), and *mcr-1* and *mcr-2* (colistin) [109]. The prophages are viral genetic materials that are located on the genome of bacteria and have also been confirmed to carry mobilisable antibiotic resistance genes [113]. These prophages are originally resident among viruses called bacteriophages, and can transfer their genome among bacteria [114]. Generally, these mobile genetic elements can transvers**e** different ecosystems and have impacted different environments in the way the**y** replicate ARGs in these different environments. The major molecular mechanisms through which these ARGs carrying MBEs disseminate into the environment are conjugation, transduction, and transformation [115]. These molecular processes take place at the different interfaces constituting the One Health, and a detailed knowledge of them is considered crucial in understanding the molecular dynamics of these MGEs and the ARGs they carry [115].

Among the major mechanisms of genetic recombination observed in the transmission of ARGs, conjugation appears to be the most common. Antibiotic resistance genes are facilitated by horizontal gene transfer, a result of closely related bacteria coming together in similar environments to exchange genes through the process of conjugation [116]. The major mobile genetic entities that facilitate the transmission of ARGs in conjugation are the plasmids. This is due to their ability to replicate independently and their possession of genes that can enhance their transfer to other bacteria. Some plasmids can carry multiple genes that allow bacteria to become resistant to different antibiotics [117]. The most common antibiotic resistance genes disseminated by HGT in different environments are those that code for extended-spectrum beta-lactamases (ESBLs). The clonality of certain plasmids that code for resistance to certain antibiotics has been established between the human population and the environment [118].

There are numerous documented cases of confirmed dissemination of ARGs from cattle and related processing facilities to the immediate environment, notably soil and water [119]. This phenomenon is more noticeable when such animals are reared intensively in an enclosed environment that requires constant washing of animal sheds with massive volume**s** of water. The fecal dissemination of antibiotic resistance genes from cattle is a paramount concern, going by the increasing intensive nature of cattle rearing to cater for the ever-growing demand for beef, milk, and other nutritional products [120]. The genes that code for resistance to third-generation cephalosporins, notably *bla*_TEM_ and *blaCTX-M*, have been found to be shed from cattle and pigs to the soil [121]. The *mecA* gene is another genetic element commonly disseminated through feces from cattle into the environment [122]. Other commonly disseminated resistance genes that occur in livestock include *tet*, which codes for resistance to tetracycline; *sul*, which codes for resistance to sulphonamides; *erm*, which codes for resistance to macrolides; and *fca*, which codes for resistance to chloramphenicol [97]). Another aspect is the carriage and global dissemination of genes that code for resistance to clinically significant last-resort antibiotics, notably the *mcr* gene that code**s** for resistance to colistin [123,124].

Due to the intrinsic nature of pigs, pig farming requires a lot of water for cooling their bodies, and the animals tend to cool off in dirty water or environments in the absence of access to clean water. This phenomenon makes pig feedlots susceptible to infections, hence the heavy antibiotic usage in pig farms [125]. Subsequently, ARB and ARGs are heavily disseminated into the surrounding agricultural soils [126]. In addition, reports have also confirmed the dissemination of ARGs, mostly tetracycline resistance genes and multidrug resistance genes, from pigs into adjoining streams and water bodies through the process of erosion or the routine runoff of water used to clean the cages used to keep the pigs [126]. The common ARGs reported in pigs and related environments include those that confer resistance to sulphanilamide (*sulI* and *sulII*), aminoglycoside (*aadA*), and tetracycline [*tet* (A) and *tet* (M)] [127].

Poultry and related products serve as major protein sources globally, and antibiotic use has become an integral practice for poultry production, especially in chickens and other birds that are reared in intensive settings [128]. There are some documented cases of feacal shedding of ARGs from poultry and wild birds [128]. Some ARGs found in the faecal matters of chickens and are disseminated into the environment include beta-lactam resistance genes *bla*_TEM_ and *bla*_CTX-M_; fluoroquinolones (*qnrA, qnrB, qnrS*); aminoglycosides (*strA-strB, aphA1, aac(3)-II*); sulfonamides (*sul1, sul2, sul3*); trimethoprim (*dfr1, dfr5, dfr7/17*); and tetracycline (*tetA, tetB*). Another major concern is the usage of last resort antibiotics in intensive poultry production and the subsequent development of resistance to such antibiotics, especially carbapenems and colistin [98,129]. The *mcr* gene is increasingly found among poultry and poultry birds and has been disseminated into immediate environments. This phenomenon further imperils the health of poultry farmers [130]. ARGs that evolve from poultry have been found in humans that have frequent direct contact with the animals. Table 1 shows a list of representative antibiotic resistance genes with One Health relevance:

**Table 1.** Selected representative antibiotic resistance genes in animals and associated antibiotics with one health relevance.

S/n	Resistance genes	Antibiotic
1	*ermB, ermF, ermT, ermX*	Macrolide (Erythromycin)
2	*tetB, tetC, tetM, tetW, tetL*	Tetracycline
3	*sul1, sul2, sul3*	Sulfonamide
4	*Cat*	Chloramphenicol
5	*blaTEM, blaCMY-2, blaCTX-M, blaSHV, blaNDM, blaKPC*	Beta Lactam
6	*qnrS1, qnrB2, qnrC, qnrD, qnrS*	Quinolone
7	*vanB, vanC1m vanA*	Glicopeptide (Vancomycin)
8	*Mcr-1, mcr-2, mcr-3*	Colistin
9	*nat, aph, aacA-aphD*	Aminoglycoside (Kanamycin)
10	*mecA, mecR1, mecI*	Methicillin
11	*VanA*	Vancoumycin

Source [131].

Some bacteria within the One Health have shown resistance to multiple classes of antibiotics. This has been attributed to their ability to accumulate different antibiotic resistance genes under the selective pressure of multiple antibiotics over time [132]. This enables such bacteria to carry different genes that code for resistance to different antibiotics, and the bacteria involved could be the same or related species. The MBEs have been notorious for the ability to carry different genes that confer resistance to multiple antibiotics, and such multiple resistance can spread in different environments that encompass the One Health [133]. Bacteria that show resistance to multiple antibiotics have been shown to emerge from farm environments, and such bacteria have been linked with those found in waterways and the human population [40].

Another important aspect of the transmission of ARGs is their dissemination at the animal-human interface. The different pathways for the transfer of ARGs from livestock feces and fecal waste waters to human pathogens have been properly delineated [97]). First, the bacteria containing the ARGs are discharged directly into the soil or water environment in proximity to the animals. The bacteria can be transmitted to humans when they colonize the gut. Within the gut, genetic elements, particularly plasmids containing antibiotic resistance genes, can be transmitted to other bacteria within the same environment through genetic recombination, notably conjugation, transduction, and transformation [134,135]. Recent advances in bacterial genomics have allowed the accurate tracking of ARGs from animals and livestock to humans [136,137]. The possible implication of the transfer of ARGs from humans to the environment is evident in the increase in the frequency of empirical treatments among humans in clinical settings, which can further increase the cost of healthcare [138]. Whole genome sequencing has also confirmed that genes can be transferred horizontally between bacteria from different environments and human pathogenic bacteria [139]. The abundance of antibiotic resistance genes in different aquatic environments is a strong consideration within the One Health framework. Intensive animal husbandry and irrigation for crops are always associated with heavy use of water [140]. A complex interplay exists in the use of water for agricultural purposes, human water consumption, and other uses of water [141,142]. However, the presence of AR organisms and ARGs in aquatic ecosystems poses a serious threat to public health [43]. The waste water originating from the hospital environment represents the largest pool of ARB and ARGs, most especially extended-spectrum β-lactamases (ESBLs) and carbapenemase-producing *Enterobacteriaceae* (CPE) [143,144].

The emergence of the carbapenemase producing enteric bacteria is a reflection of the heavy use of the third generation cephalosporins and the carbapenems as clinically relevant antibiotics that should only be limited to clinical environments. Hospital effluents have been known to be released into nearby aquatic environments that are further polluted with antibiotic resistant genes [145]. Aquaculture also makes use of antibiotics to increase productivity and contribute significantly to global protein needs [146]. This leads to the emergence of ARB carrying different ARGs. Among the antibiotics used in aquaculture, the quinolones, tetracyclines, chloramphenicols, and sulfonamides are prominent [146,147]. Examples of genes that have been found in aquaculture include *qnrA* and *qnrS* genes that confer resistance against fluoroquinolones [148] and broad-spectrum β-lactamase resistance genes, including *blaTEM-52* and *blaSHV-12*. A study by Delannoy et al. [149] highlighted the presence of antibiotic resistance genes that included *ul-1*, *ant (3′′)-Ia*, *aph(3*′*)-Ia*, *strA*, *strB*, *dfrA1*, *qnrA*, and *bla*_CTX-M-9_ among different Gram-negative bacteria isolated from fishes and shrimps in aquaculture. Some of the genes can be located on the chromosome or on plasmids and mobile genetic elements that facilitate their transfer to pathogenic bacteria that can ultimately infect humans.

The use of manure from different animals is a cheaper alternative to fertilizers on farms, especially in Africa and Asia. The use of animal faeces as manure in agriculture to improve the yield of crops introduces ARB and ARGs directly to the soil. Studies have confirmed that animals’ faeces contain residual concentrations of antibiotics due to the incomplete metabolism of chemicals in animals. The residual concentrations of antibiotics introduced to the soil through manure create a selective pressure that accelerates the emergence of ARB in the soil [150]. The ARB, with their resistance genes, can be transferred into adjoining water bodies through erosion and runoff. ARGs in aquatic ecosystems and agricultural waters have been shown to be more easily picked up by bacteria in aquatic environments than those found in the soil [151]. Mobile genetic elements and plasmids harbouring ARGs have also been found in aquaculture and other aquatic environments. This increases the possibility of the transmission of these genes into other ecosystems and eventually to humans. A study by Lassen *et al*. [152] discovered a total of 160 ARGs in commercial aquaculture systems in Bangladesh. The common antibiotic resistance genes recovered in aquaculture systems include *sul1*, *sul2*, *bla*_CMY_, *bla*_OXA_, *qnrS*, *tetW*, *tetQ*, *tetM*, and *intl1*. Some of these genes have been recommended for standard surveillance of ARGs in water systems and aquaculture [153]. Different metagenomics approaches have revealed that certain ARGs are shared and exchanged between the gut microbiomes of humans and animals [154]. Animal feeds are also considered a repository of ARGs from where transmission to humans has been reported [155]

There is evidence that different ARGs can be transmitted from poultry and swine dung to the soil and further to adjoining aquatic environments. According to some studies, the faeces of poultry and pigs carried more antibiotic resistance genes compared with the faeces of cattle and sheep. Furthermore, some studies have reported that the abundance of most ARGs and bacterial pathogens was significantly increased by the application of composted manure, especially in surface soil [156]. Several ARGs have been found in animal manure. These genes include *ermB*, *ermF*, *ermT*, *ermX, tetA/C*, *sul1*, and *sul*2 genes [131]. Similar investigations revealed that aminoglycoside and sulfonamide resistance genes (*aac* (6’)-Ib, *aad*A, and *sul*1 are common among manure impacted soils and may be affected by levels of some metals in the soil. Organic fertilization of farmlands is found to increase the relative abundance of resistance genes compared with inorganic fertilization [157]. Mobile genetic elements being present in manure samples and their relative abundances have also been noted to increase the chances of transmission of antibiotic resistance genes in manure environments (Yuan *et al.*, 2022). The most common mobile genetic element is the class I integron, which is common in pig manure [158]. It has also been reported that there is a great diversity in the genetic elements and insertion sequences that are found in manure and wastewater treatment plants. The major concern, however, is the abundance of mobile genetic elements, which also have a positive influence on the abundance of ARGs that can be found in manure, soil, and aquatic ecosystems. The resistome is the entire set of ARGs within a particular environment, and it provides insights into the diversity of antibiotic resistance genes within particular environments as well as the transmission patterns of the genes between different environments and ecosystems. The emerging field of metagenomics is also adding another dimension to our understanding of the antibiotic resistome that exists in different environments and ecosystems. It facilitates a deeper understanding of the complex interaction between the resistome of different environments from a One Health perspective.

The interconnected nature of the One Health implies that the resistome in different environments can be correlated to access the pattern and potential transfer of antibiotic resistance genes between different components of the One Health. The air resistome increases the risk of transmission of ARGs through aerosolized gene transmission to humans [159]. The relations between the resistomes of different environments can be established with the overlap of the bacteriome and resistomes recovered from the different components within the One Health framework [160]. Considerable resistome overlap has been detected within the gut of humans and animals as evidence of the interchange of antibiotic resistant genes between these entities [161]. A resistome diversity study carried out by Lawther *et al*. [162] reported that there was substantial overlap among the resistomes of livestock, poultry, swine, and soil, with tetracycline (*tet*) resistance genes as the most frequently reported gene common to all the resistomes. This points to the potential dissemination of resistance genes between different animals and the soil ecosystem.

## Conclusion and recommendation

The concept of One Health has been recognized as an interdisciplinary entity that comprises human, animal, and environmental ecosystems and how they can be harnessed as an entity to address important public health problems. Some countries have integrated One Health into their public health systems, but it appears the impact of this inclusion is not yet under consideration [163]. It is important that programs be established to monitor the implications and impact of One Health policies in countries that have started using the approach. The monitoring of the impact of the various One Health programs will likely unravel certain aspects that need to be improved in order to solve the different public health problems targeted by the programs, including antibiotic resistance. The problem of ARBs and resistance genes in the environment has been recognized as an environmental problem, in addition to the challenge they pose to public health [30]. Several studies are increasingly focusing on ARB and resistance genes as environmental problems, and this approach is increasing the understanding of the environmental aspects of antibiotic resistance. Many such studies have been reported in developed countries [164].

The emergence of antibiotic-resistant bacteria within farm animal**s** and wildlife has serious implications to public health, the ecosystem, and policy. The deluge of such findings will prioritize mitigating factors that will contribute to reducing animal use of antibiotics in order to preserve the efficacy of such antibiotics, especially those that have direct clinical relevance for human health [165]. The presence and abundance of ARGs in water systems and adjoining ecosystems will help prioritise the deliberate effort to reduce the abundance of antibiotic resistance genes in water and sewage and help improve on existing systems and technologies that are currently in use to remove antibiotic resistance genes.

The use of manure from different sources in agriculture is an aspect that should be critically evaluated, especially in low and middle income countries. This has become a regular practice, partly due to the lower cost and easy access for local farmers. The best approach could be to assist with technologies to pre-treat manures to remove ARB**s** and ARGs before they are used for different purposes in agriculture. The immediate release of feces into waterways and the environment is also a common practice, and their pre-treatment will limit the risks of direct dissemination of ARGs into the environment. The pre-treatment of manure has proven to be effective in reducing the concentration of residual antibiotics, thereby reducing the risk of the evolution of ARB and their associated genes [166]. The pre-treatment of wastewater before release into municipal drinking water sources is also a standard practice in developed countries, but unfortunately, such technologies and expertise are not common in developing countries. This is another measure that can be used to limit the emergence of ARB, dissemination of ARGs and further limit the associated risks to public health.

One Health is a comprehensive strategy that incorporates the different entities of human health, animal health, and the environment. One Health studies should include expertise in these different entities to properly synchronize their different effects on antibiotic resistance. Isolated studies that incorporate only one of these entities may not be effective enough to ameliorate the problem of ARB and resistance genes. Different countries needs to further improve on their One Health strategies to ensure that they are incorporated ant practised at regional and sub-regional levels. This will ensure that such strategies have the maximum impact towards preventing AR and improving public and ecosystem health.
